# Implementing a community-level intervention to control hypertensive disorders in pregnancy using village health workers: lessons learned

**DOI:** 10.1186/s43058-020-00076-8

**Published:** 2020-10-02

**Authors:** Olukolade George Shobo, Anselm Okoro, Magdalene Okolo, Peter Longtoe, Isaac Omale, Endurance Ofiemu, Jennifer Anyanti

**Affiliations:** Society for Family Health, 8 Port Harcourt Crescent, Area 11, Garki, Abuja, Nigeria

**Keywords:** Community health workers, Hypertension in pregnancy, Resource-poor setting, Primary healthcare

## Abstract

**Introduction:**

Hypertensive disorders in pregnancy result in about 76,000 maternal deaths per year worldwide. Pre-eclampsia and eclampsia cause the most deaths. Interventions for managing these disorders are available in health facilities. We assess the effect of monitoring pregnant women’s blood pressure (BP) in their homes using village health workers (VHWs) equipped with a BP-measuring device on hypertension in pregnancy, in a resource-poor setting. Also, we assess the VHWs’ competence with the BP device, acceptability and appropriateness of the intervention, and factors that affect the implementation of the intervention.

**Method:**

This is a mixed method study comprising quantitative and qualitative data collection. We implemented the intervention over 6 months across three local government areas in Gombe state, northeast Nigeria. The Replicating Effective Program (REP) framework guided the development of the implementation strategy. The quantitative data include routine measurement of pregnant women’s blood pressure and observation of 118 VHW-client interactions. The routine data collection occurred between February and June 2019, and the observation occurred in January and June 2019. The qualitative data collection occurred via six focus group discussions with VHWs and ten in-depth interviews with community health extension workers in June 2019. We analyzed the data from the quantitative arm with SPSS version 23. For the qualitative arm, we transcribed the audio files, coded the texts, and categorized them using thematic analysis.

**Result:**

Nine thousand pregnant women were recruited into the program. We observed a significant reduction in the prevalence of hypertension in pregnancy from 1.5 to 0.8% (*Z* = 4.04; *p* < 0.00001) after starting the program. Also, we found that VHWs can assess pregnant women’s BP using a semi-automatic BP-measuring device. The intervention is acceptable and appropriate in resource-poor settings. Poor payment of VHW stipend and cooperation of local health staff are barriers to sustaining the intervention.

**Conclusion:**

In resource-poor settings, health systems can train and equip non-technical people to identify and refer cases of high blood pressure in pregnancy to local health facilities on time. This may contribute to reducing maternal mortality and morbidity in these settings.

Contribution to the literature

- Our study is the first to provide evidence on the implementation approach and result of using level 1 community health workers equipped with a blood pressure-monitoring device to partake in managing hypertension in pregnancy in resource-poor settings.
- We implemented the intervention in a real-world setting that has a weak health system that serves socioeconomically disadvantaged populations.
- The findings show that health systems in resource-poor settings can increase the coverage of life-saving interventions for pregnant women by implementing strategies that shift tasks, such as blood pressure measurement, referral, and follow-up services, to non-technical women in rural communities with poor access to care.

## Introduction

Hypertensive disorders affect 10–15% of pregnancies [1], resulting in about 76,000 maternal deaths per year across the world [2, 3]. Ninety-nine percent of these deaths occur in low- and middle-income countries (LMICs) [4]. Of these disorders, pre-eclampsia and eclampsia cause the most deaths [5–7]. Pre-eclampsia is hypertension and proteinuria with or without pathologic edema, occurring after 20 weeks of gestation [8–10]. The American College of Obstetrics and Gynecology does not require proteinuria to define pre-eclampsia if end-organ failure exists [8]. If left untreated, pre-eclampsia progresses to eclampsia which can threaten the life of the mother and the unborn child [2, 9, 11]. Eclampsia is the new onset of a grand mal seizure with or without unexplained coma in a woman with pre-eclampsia [12]. It may turn up in a patient with minimal elevation in blood pressure and no proteinuria [10].

Effective interventions for managing pre-eclampsia and eclampsia exist. They include calcium for pre-eclampsia prevention, risk stratification, and antihypertensive and magnesium sulfate therapy [13, 14]. These interventions are healthcare facility-based [14]. Even with high coverage of these interventions in health facilities, it does not mean that maternal deaths will be reduced [14, 15]. This is because in LMICs, close to 50% of eclampsia cases first convulse in the community before they can reach adequate care [16]. Close to 7% of pregnant women with eclampsia who live within the catchment area of a health facility with intensive care still die [14]. The shortage of healthcare workers, delays in early disease identification, and poor access to care are still challenges to the effective coverage of these interventions in LMICs [16, 17].

Early identification and sorting of cases of pre-eclampsia and eclampsia can avert close to 50% of maternal and fetal deaths that occur because of the diseases [18–20]. Innovative approaches to improve the demand for and coverage of health facility-based interventions for pre-eclampsia and eclampsia are needed [13, 19]. One way forward is to employ a task-shifting approach [16] and use community health workers (CHWs) to screen, identify, and refer pregnant women needing these interventions to the local health facilities [19, 21]. Community health workers are members of the community, selected, trained, and working as health aides in communities, either for pay or as volunteers, with the local healthcare systems [22, 23].

Based on the duration of their training, CHWs are categorized as level 1 or 2. Level 1 CHWs receive training for 8–21 days and are resident in the community. Their local health systems expect them to provide most of their services through community-based activities such as home visits and community outreaches. This cadre of CHWs includes village health workers [22]. Level 2 CHWs have post-secondary pre-service training in a government-accredited institution lasting anywhere between 3 months and 3 years. They are healthcare facility-based, relying on women and babies to access services at the healthcare facility they work. They conduct occasional home visits and community outreaches. This cadre of CHWs includes community health extension workers (CHEWs) [24]. In many LMICs, CHWs already exist and deliver maternal and newborn health services in their communities and refer cases needing a higher level of care [19, 22, 24]. Some services they offer include the provision of safe delivery services for pregnant women and health education on non-communicable diseases [22, 25]. While the role of CHWs does not include managing pregnancy conditions like pre-eclampsia and eclampsia [26, 27], the evidence suggests that level 2 CHWs can detect abnormal blood pressure and treat the diseases in some LMICs [27–30].

In 2019, the Society for Family Health in collaboration with Gombe state government in Nigeria started the first phase of a state-wide community-level management of hypertensive disorders in pregnancy using VHWs. They equipped VHWs with skills and tools for the work. Also, level 2 CHWs were equipped with skills and tools to supervise the VHWs. To the best of our knowledge, the literature around the use of level 1 CHWs for assessing, monitoring, and referring cases of hypertension in pregnancy using a blood-pressure monitoring machine is lacking. In this paper, we describe the intervention, its implementation, and the result guided by the Standards for Reporting Implementation Studies (StaRI) guidelines [31] and the Consolidated Criteria for Reporting Qualitative Studies (COREQ) checklist [32]. We have three research questions. First, can VHWs in resource-poor settings assess pregnant women’s BP to produce improvement in those that are hypertensive during that pregnancy? Second, in what way does the community respond to this kind of intervention? Last, what factors affect the sustainability of this intervention? Our aim is to investigate the acceptability and appropriateness of the intervention, the competence of VHWs with the BP machine, the factors that affect the implementation, and the effect of the intervention on the prevalence of hypertension in pregnancy. We believe our study will contribute to the body of knowledge and discourse about developing and scaling-up of community-level interventions for managing hypertensive disorders in pregnancy using non-technical and/or low-cadre healthcare workers in LMICs.

## Method

### Study design

This is a mixed method study comprising quantitative and qualitative arms. We implemented both arms independently, to derive a broader interpretation of the intervention’s result [33, 34]. The integration of the two arms occurred during the analysis and interpretation of the findings [35, 36]. The quantitative arm included routine measurement of all pregnant women’s blood pressure in target communities and observation of 118 village health worker (VHW) interactions with their clients. We collected the routine data between February and June 2019 and the observation data at two time points—first in January 2019 and then in June 2019.

The qualitative arm comprised six focus group discussions with VHWs and in-depth interviews with ten community health extension workers (CHEWs) in June 2019. Trainings for the VHWs and CHEWs and follow-up refresher trainings for the VHWs associated with the study occurred over 5 weeks between December 2018 and January 2019, before any data collection.

### Study context

The intervention is part of a broader VHW program implemented by the Society for Family Health and Gombe State government across 57 of 114 political wards in the state [37]. Gombe state is in northeast Nigeria. It has 11 local government areas (LGAs), 114 political wards, and three senatorial zones [38]. The population of the state is about 3.4 million, and the fertility rate is 7.3 [39]. About 23% of the women age 15–19 have begun childbearing, and 14% of all women are pregnant at any point in time [40, 41]. There are 603 health facilities across the 11 local government areas in the state. Close to 90% of these health facilities are public PHCs. Fewer than half of pregnant women in the state access pregnancy care at least four times as recommended. A much smaller fraction access facility-based intrapartum care and/or skilled attendance at birth, and close to 40% do not have their blood pressure assessed at all during pregnancy [42, 43].

#### The VHW program

**Fig. 1:**
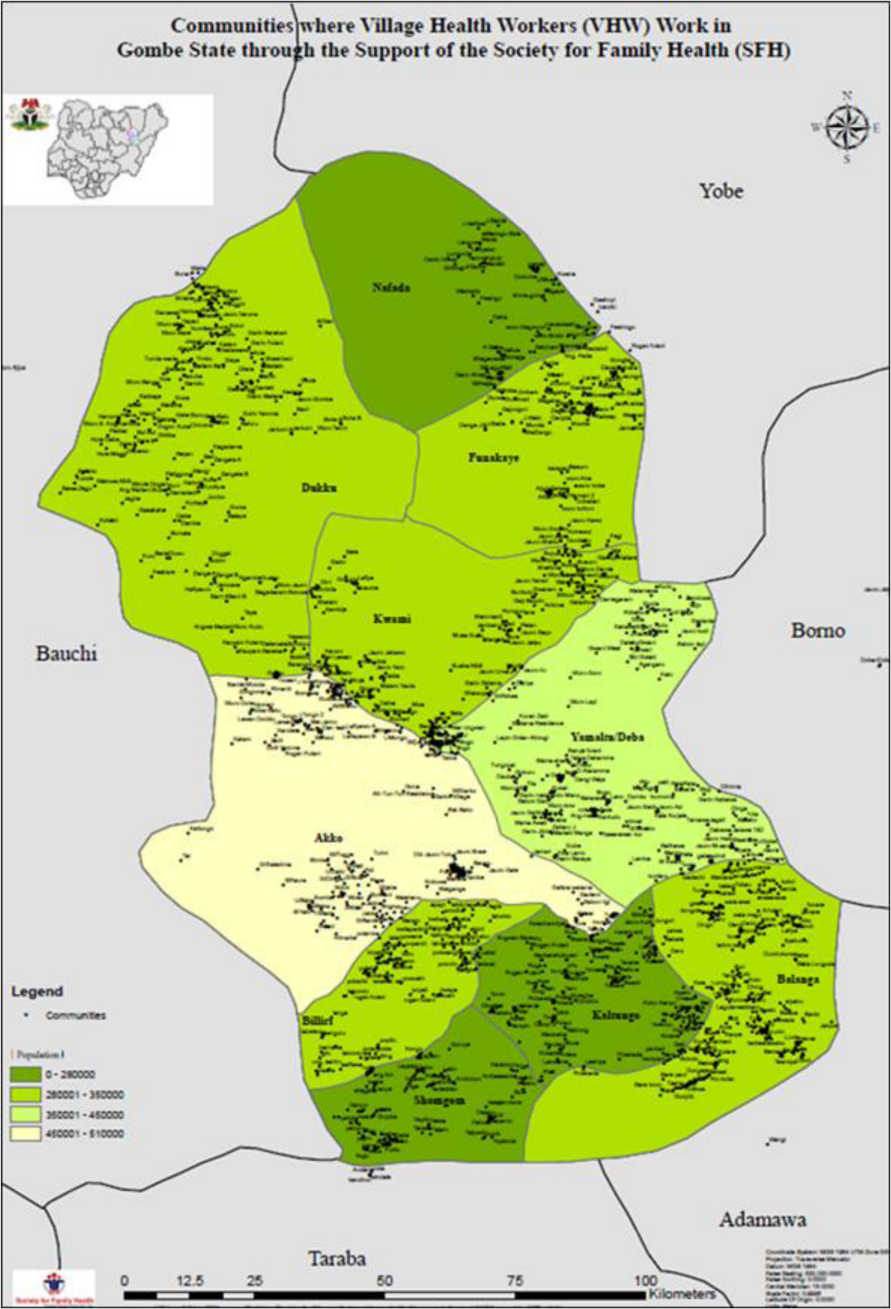
Communities VHWs cover in Gombe state

In Gombe state, the appellation for level 1 CHWs is village health workers [22]. They are members of the communities they live and work in. Also, they are answerable to their communities and supported by the Gombe State Primary Health Care Development Agency. We trained the VHWs for 21 days to deliver community-level maternal and newborn health promotion services; offer prophylactic medications such as misoprostol and chlorhexidine to clients who live very far from any health facility, who deliver at home, and experience postpartum hemorrhage; generate the demand for health facility-based maternal and newborn health (MNH) care; and refer or accompany MNH emergencies to health facilities. The training was led by the government. There are 1200 VHWs in Gombe state working in 2000 communities across the 11 LGAs in the state (Fig. 1). Most have at least a secondary school education and can read and write in English or Hausa. The VHWs visit pregnant women in their homes at least four times during pregnancy and twice in the postnatal period. Trained CHEWs in the local primary care clinics supervise the VHWs. These trained CHEWs in turn train their colleagues in the health facilities. The CHEWs and their colleagues participate in the care of clients and patients referred by the VHWs. Each VHW receives a stipend of about $17 per month from the government.

### Target sites

Implementation of the intervention occurred in Gombe, Akko, and Balanga local government areas (LGAs), in Gombe state. These LGAs had the highest number of VHW visits to pregnant women, 6 months prior to starting the intervention in each of the three senatorial districts of the state. Gombe LGA is the state capital, located in the Central Senatorial Zone. It has 140 VHWs working across five of the ten political wards in the LGA. It has an area of 52 km^2^ and density close to 7100/km^2^. Akko LGA is in the North Senatorial Zone and has 154 VHWs working across five of the 11 political wards in the LGA. It has an area of 2627 km^2^ and a density close to 177/km^2^. Balanga LGA is in the South Senatorial Zone of the state and has 118 VHWs working across five of the 10 political wards in the LGA. It has an area of 1626 km^2^ and a density close to 180/km^2^ [44]. The three LGAs have a total population of about 1.1 million. We estimated that there will be close to 2300 pregnant women per month across the 15 wards the VHWs work [45]. All pregnant women in the 15 political wards were eligible for the intervention.

### Intervention strategy

We established the routine measurement of pregnant women’s blood pressure (BP) in rural and hard-to-reach communities using VHWs equipped with the Microlife CRADLE VSA BP measuring device. To deliver the intervention, we added the BP assessment to the work the VHWs already do. We assumed that the acceptability of the VHWs in their communities will make the add-on intervention acceptable to the communities. The VHWs were supervised by trained CHEWs. They referred cases with abnormal BP to the local public health facilities where the CHEWs work as part of the intervention.

Though VHWs visit a pregnant woman at least four times during pregnancy, our intervention focused only on the first two visits. Time and budget constraints did not allow for longer follow-ups during the study. The VHWs commenced home visits as soon as a pregnant woman was identified in the community, irrespective of her gestational age. A VHW’s first BP assessment occurs during the first home visit to a pregnant woman. The second BP assessment occurs during the VHW’s second home visit. The VHWs schedule the second home visit to occur 4 weeks after the first. During the visits, the VHW offer the pregnant woman health education around BP disorders in pregnancy, what they mean, and when to seek help.

When a VHW assesses a pregnant woman, if an abnormal BP reading is gotten on the measuring device, the pregnant woman is allowed to rest for 15 to 20 min before a repeat measurement is carried out. If the indicator light is different after the repeat measurement, the VHW allows the pregnant woman to rest for another 5 min and repeat the measurement the third time. The third measurement is the tie-breaker. The VHW records the BP reading of the repeat measurement and takes action by referring the woman to a health facility if needed. They also provide health advice using information education communication tools designed by the Society for Family Health as appropriate. We have further described the details of the intervention elsewhere [46].

#### The Microlife CRADLE VSA device

The Microlife CRADLE VSA is a hand-held BP monitoring device. It has a traffic light indicator and screen that shows systolic and diastolic pressures [46–48]. When the device shows a green light, it means the blood pressure of the pregnant woman assessed is normal. This means the systolic blood pressure is at least 100 mmHg but less than 140 mmHg, and the diastolic blood pressure is at least 60 mmHg but less than 90 mmHg. A yellow light means the pregnant woman needs a referral to a health facility. In this situation, the systolic blood pressure is at least 140 mmHg or the diastolic blood pressure is at least 90 mmHg. When the indicator is yellow, the pregnant woman must be referred within 24 h of being assessed. A red light suggests severe hypertension or shock. It means the systolic blood pressure is at least 140 mmHg, and the diastolic blood pressure is at least 90 mmHg. In this situation, the pregnant woman should get moved to the health facility right away. The device is valid for assessing BP in pregnancy and pre-eclampsia. It has also fulfilled the standard required by the British Hypertension Society (BHS) grading criteria, Association for the Advancement of Medical Instrumentation (ANSI/AAMI/ISO), and World Health Organization (WHO) for low-resource settings [49, 50].

### Implementation strategy

We implemented the intervention for 6 months. First home visits occurred for the first 4 months. The second visit started from the second month to the end of the implementation period. The Replicating Effective Program (REP) framework guided the development of the implementation strategy. The REP framework has been used to develop and disseminate effective HIV interventions across the world. Its key components crucial to implementing effective interventions in healthcare are intervention packaging, training, technical help, and fidelity assessments [51]. We present the implementation strategy in Table 1.

**Table 1:** Community-level strategy for controlling hypertensive disorders in pregnancy using existing VHWs equipped with the Microlife VSA device

Strategy	Engage government to take ownership and leadership of the initiative	Build local capacity to implement and scale up the BP intervention through existing community health programs	Monitor pregnant rural women’s blood pressure and refer cases to health facilities	Supportive supervision for VHWs
Actors	Investigators, implementers, and leadership of GSPHCDA	Consultant from teaching hospital, implementers	Village health workers	CHEW supervisors
Actions	*Pre-implementation meetings* on rationale *Official notification of LGA PHC offices and community structures of governance* via the leadership of GSPHCDA. *Procurement of CRADLE Micro VSA BP device* by SFH. *Engagement of consultant* for trainings	Training workshop for: *-* 22 master trainers *-* 15 CHEWs *-* 412 VHWs CHEWs further trained on supportive supervision On-the-field refresher trainings for VHWs 4 weeks after the first training	Identify pregnant women in the community Monitor their blood pressure during scheduled home visits Refer pregnant women with abnormal blood pressure to a health facility	Supportive supervision to VHWs 1st week post-training Meetings with VHWs at least once a month to assess progress with implementing the intervention and address data management issues
Target	Government leadership and ownership of the intervention Readiness to use intervention’s result for decision-making	Pool of local experts with the capacity to scale up or the intervention in the state VHWs competent in assessing pregnant women’s BP and offering appropriate referral advice	Improve coverage and access to quality obstetric services in hard-to-reach communities	Continued delivery of quality community level obstetric services through VHWs
Temporality	Step 1	Step 2	Step 3	Step 4
Dose	As needed	Two-day 16-h training for 22 master trainers Two-day 12-h training for VHWs and CHEWs in clusters of 40 participants per class Two extra hours training for CHEWs Three hours on the field refresher training for VHWs in clusters of five to seven participants	At least two new home visits per week Revisits occur within 4 weeks of the first visit	1st supportive supervision done in the 1st week post-training, then once for each VHW in the first month after the training Then once a week for VHWs identified as having challenges
Implementation outcomes addressed/affected	Adoption Capacity of government lead implementation Readiness of government to scale up the intervention	*Master trainers*—availability of local resource persons for scale-up of the intervention *Master trainers, CHEWs, and VHWs* awareness and knowledge of hypertension in pregnancy and management, using the Microlife VSA device *VHWs* Capacity to assess pregnant women’s BP and refer abnormal cases to a health facility using the Microlife VSA device Capacity of CHEWs to ensure fidelity of the implementation strategy	Penetration and acceptability of the intervention Productivity of VHWs	Quality of services delivered Fidelity of the implementation strategy
Theoretical framework	Replicating effective programs (REP) framework for healthcare interventions			

Based on framework suggested by Proctor et al and Pinnock et al [31, 50]

### Outcome measures

Our outcomes follow the implementation and intervention strands. We describe the primary and secondary outcomes for the two strands in Table 2. For the implementation outcomes, acceptability is respondents’ perception on whether the innovation is agreeable to beneficiaries and stakeholders. Appropriateness is the perceived fit of the innovation for the setting. Competence is the ability of VHWs to perform expected tasks with the BP device and understand its readings [52]. We assess acceptability and appropriateness under the qualitative arm of the study, and competence via observing the VHW-client interactions.

**Table 2:** Outcomes for implementation and intervention strategy

Outcomes	Implementation strategy	Intervention strategy
**Primary**	• Acceptability of the intervention • Appropriateness of the intervention	• Prevalence of hypertension in pregnancy
**Secondary**	• Competency of VHWs with use of BP device *- % VHWs that can properly apply the BP device’s cuff* *- % VHW that identify the systolic and diastolic readings on the device* *- % VHWs that can interpret the traffic colors on the device* *- % of VHWs that can define the error messages on the device*	• Prevalence of isolated systolic hypertension in pregnancy • Prevalence of isolated diastolic hypertension in pregnancy

For the intervention outcomes, hypertension is blood pressure equal to or higher than 140/90 mmHg [53]. Hypotension is blood pressure that is lower than 90/60 mmHg [54]. Isolated systolic hypertension is systolic BP equal to or above 140 mmHg in the face of a normal diastolic BP [55, 56]. Isolated diastolic hypertension is diastolic blood BP above 90 mmHg with systolic pressure being normal [57]. We assess the intervention outcomes with the BP data collected by VHWs during the home visits.

### Study population and sampling

#### Quantitative arm

##### Routine BP measurement by VHWs

We recruited all consenting pregnant women in the target sites in the study. The VHWs used an active house-to-house search and snowball sampling technique to find pregnant women.

##### Observation of VHW-client interaction

The 412 VHWs across the three target LGAs form our study population. We selected all 140 (34%) VHWs in Gombe LGA for observations using a purposive sampling technique. Observations of the same set of VHWs occurred at the baseline and end line period. Budget and logistic constraints informed the choice of the sampling technique used. The geographic spread of VHWs in the other LGAs made the observation in these LGAs a challenge.

#### Qualitative arm

We recruited 57 VHWs and conducted six focus group discussions (FGDs)—two in each of the LGAs. The FGDs had eight to ten respondents each. We worked with the CHEWs who supervised the VHWs to select respondents for the FGDs across the three LGAs using a purposive sampling technique. We assumed that the VHWs selected could give rich information about the intervention and describe the significance of their experience.

Also, we carried out in-depth interviews (IDIs) with 10 of the 15 CHEWs that supervised the VHWs.

### Data collection

#### Quantitative arm

##### Routine BP measurement by VHWs

We trained the VHWs to collect data on a routine basis during home visits to pregnant women. The VHWs entered the BP readings into a register and a hand card which they gave the pregnant woman during their first visit. The information entered in the register included the date of visit, whether the visit was antenatal or postnatal, the health education topic the VHW offered the pregnant woman during the visit, health facility-based obstetric services received by the pregnant woman prior to the VHW’s visit that day if any, BP readings from the Microlife CRADLE device, symptoms of illness or danger signs of pregnancy, and whether the pregnant woman got referred during the visit. The VHWs submitted the registers for 2 days every month to the program officers at the Society for Family Health. The program officers enter the data from the registers into an MS Excel spreadsheet. The CHEWs ensured data quality by conducting a weekly review of the VHWs’ registers and supportive supervision during selected home visits. The CHEWs focused on ensuring the completeness, reliability, and validity of the routine data collected.

##### Observation of VHW-client interaction

We recruited and trained observers to collect observation data during VHW-client interactions. A checklist of steps for assessing pregnant women’s BP with the Microlife CRADLE device was used for observation [58]. It included items around the proper application of the device’s cuff on the pregnant woman’s arm, when the inflation bulb of the Microlife CRADE device is pumped, when the pumping of the inflation bulb is stopped, interpretation of the reading of the device and the color code after assessing the pregnant woman’s BP, and the documentation of the client’s data and BP reading into a mock register similar to the one they used during home visit. They also asked the VHWs to define the error messages the BP device is known to give after each observation.

The first data collection occurred in the period after the workshop trainings and before the refresher trainings for VHWs on the field occurred. We describe this period as the baseline period. The second data collection occurred at end of the study period in June 2019. The observations occurred in five local primary health facilities each situated in the wards in which the VHWs worked. We chose the antenatal clinic days in these health facilities for observation to improve the chances of VHW-client interactions. Data collection occurred in identified private areas of the health facilities in front of the observers. The ratio of VHW-client interaction observed was 1 to 1. Observations occurred in a health facility because the cost for observing VHWs during home visits was inhibitive. The trained observers only corrected the VHWs on mistakes made after the observation was over. The same VHWs got observed during both time points.

#### Qualitative arm

Trained interviewers carried out semi-structured interviews using prepared interview guides. Data collection occurred at the end line of the study. They used a recording device during all interviews. Also, they got consent from the respondents before the interviews and recording started. Transcribers took notes during the interviewers. The interview guide for the FGDs included questions on VHWs’ confidence with using the BP device; ease of learning how to use the device; acceptability of the innovation, appropriateness of the implementation approach, perceived impact; and implementation challenges. The interview guide for the in-depth interview included questions on perceived and observed competency of VHWs with the use of the CRADLE device, challenges with implementation, acceptability of innovation in the health facility, and perceived impact of the intervention. Trained supervisors monitored the interviews conducted. All qualitative interviews occurred at the end line of the study period.

### Data analysis

In this section, we describe the analysis used for each arm of the study. They are as follows:

### Quantitative arm

#### Routine BP measurement by VHWs

We cleaned and coded the data in MS Excel and used SPSS 23 for the analysis. For each of the two VHWS visits under the study, we categorized the pregnant women’s blood pressure measurement using the American Heart Association classification. We then used descriptive statistics to show the proportion of pregnant women in each of the categories and the corresponding confidence intervals for the proportions. Also, we conducted *Z* tests to compare the significance of the difference between the proportions for the two time periods, after conducting a test of normality. We set the confidence interval and significance level for all the tests at 95% and 5%, respectively. All measurements from the first and second visits were included in our analysis. We did not discard the data of pregnant women who received a first visit but not the second visit.

#### Observation of VHW-client interaction

We used descriptive statistics to describe how VHWs that could perform specified tasks with the CRADLE device at baseline and end line are distributed. Also, we used McNemar’s chi-squared test to analyze whether the proportion of VHWs who could not apply the BP device on pregnant women (as opposed to those who could) decreased by end line, following the on-the-field refresher trainings. The confidence interval and significance level were set at 95% and 5%, respectively.

### Qualitative arm

We transcribed the audio data from the FGDs and IDIs in full and analyzed them using Saturate® at http://www.saturateapp.com/—a free online qualitative data analysis tool. First, two people from the team each analyzed the subsets of the datasets. They generated separate coding frameworks for the datasets. They then deliberated and agreed on the final coding frameworks for the two—FGD and IDI—datasets. We used the final coding frameworks to analyze the rest of the dataset. After coding the rest of the datasets, we then categorized the codes under themes using an inductive approach. The Consolidated Criteria for Reporting Qualitative Research [32] guided our qualitative analysis approach.

### Ethics

We got approval for the study from the Ethical Review Committee/Board of the Gombe State Ministry of Health—Ref: MOH/ADM/S/658/Vol. II/105. We also obtained administrative approval from the Gombe State Primary Health Care Development Agency.

## Results

### Study participants

#### Routine BP measurement

Between February and May 2019, the VHWs assessed the BP of 9000 pregnant women out of an estimated 9170 across the study sites, bringing the coverage of the intervention to 98%. Of the 9000 pregnant women assessed, 56% (5024) reported their ages. The remainder could not estimate their ages even when probed with local and national historical milestones. The mean age was 25 years (SD = 5.5 years). Twenty-eight percent of the pregnant women were first reached in February, 26% first reached in March, 24% reached in April, and 22% first reached in May 2019. Also, of the 9000 reached, 40% were from Akko, 23% from Balanga, and 37% from Gombe LGA (*χ*^2^ = 487.95; *p* < 0.001). Last, 6992 (78%) of these 9000 pregnant women received a second VHW visit between March and June 2019 (Table 3).

**Table 3:** Distribution of pregnant women assessed by VHWs

Pregnant women reached with the intervention	%, *n* = 9000	Test (*p* value)
Age = 25 years	NA	NA
SD = 5.5 years		
Where they lived		
Akko LGA	40	*χ*^2^ = 487.95 (< 0.001)
Balanga LGA	23	
Gombe LGA	37	
When first visit occurred		
February	28	*χ*^2^ = 81.5 (< 0.001)
March	26	
April	24	
May	22	

The mean age of the 412 VHWs that collected the data was 30 years (SD = 7.2 years), and 76.9% (317) had a senior secondary school certificate. Also, 10% (41) had a diploma in health technology, 1.7% (7) had a diploma in education, and 0.2% (1) had tertiary education. Only 10.4% (43) had only a primary school leaving certificate. Ninety-five percent (393) were married, and the remaining 5% (19) were either divorced, single, or widowed (Table 4).

**Table 4:** Characteristics of VHWs that implemented the intervention

Application of CRADLE device on clients: VHWs observed	Frequency	Percent
Mean age = 30 years	NA	NA
SD = 7.2 years		
**Educational level**		
Islamic studies	1	0.2
Primary	43	10.4
Junior secondary school certificate	2	0.5
Senior secondary school certificate	317	76.9
School of health technology	41	10
National college of education	7	1.7
Higher (tertiary) degree	1	0.2
**Marital status**		
Single	10	2.4
Married	393	95.4
Divorced	1	0.2
Widowed	8	1.9

#### Observation of VHW-client interaction

Of 140 (100%) VHWs in Gombe LGA, we observed 118 (85%) at baseline and end line. The mean age was 29.6 years (SD = 6.3 years), and 99% (117) had some senior secondary school education. Also, 95% (112) were married, 95% (112) had at least one child, and 73% (86) did not have any other occupation apart from being a VHW. Forty percent (48) identified themselves as Fulanis, and 93% (110) said they are Muslims.

#### Qualitative arm

Fifty-seven (100%) VHWs took part in six FGDs. Their mean age was 32.3 years (SD = 6.7 years), and 84% (48) of them had at least some senior secondary education. Ninety-seven percent (55) were married, 70% (40) had no occupation apart from being a VHW, 56% (32) were Christians, and 45% (26) from the Waja tribe. Also, 10 (100%) CHEWs took part in the IDIs. Their mean age was 39.9 years (SD = 4.5 years), and 90% were married (90%). Sixty percent (6) of the CHEWs identified as Muslims.

### Acceptability

The qualitative findings indicate that the innovation is agreeable with the pregnant women in the setting. The VHWs observed improved reception during their home visits associated with the addition of the innovation to their work.

No one has rejected me or any other village health worker I know. They (pregnant women) even follow us to our houses to check their blood pressure. Even when they have a slight headache, they come knocking (at my door) to have their BP checked. As long as you are at home, one may get four to five pregnant women coming to ask to check their BP. VHW1

The VHWs also reported that the husbands of the pregnant women accept the innovation and actively demand for it.

If I don’t visit a pregnant woman as scheduled, her husband will ask me if everything is ok when he sees me around in the community. He’ll remind me to please check on his wife. This never used to happen before. They (pregnant women and husbands) welcome us more warmly now and are happy to see us in their homes. VHW2

### Reason for acceptability

Some VHWs perceive that the acceptance of the innovation is associated with its availability, ease of access, and opportunity cost of going to the local hospital to get a similar service.

They (community) are very happy because help has reached their doorstep. Before, they go the hospital and come back late, now they are being taken care of at home. VHW1

The CHEWs however suggest that while the intervention is acceptable, the implementation approach is not acceptable in full by some healthcare workers in the local hospitals where they work. They believe this is because these colleagues believe the supervisors are paid extra to oversee the implementation of the intervention.

some of them (our colleagues) think we are being paid. When a pregnant woman comes to the hospital for her antenatal visit and she presents the hand card (given by VHWs) … they won't fill or sign it. They will tell her to wait for us…the ones who attended the training and got per diem(from the training workshop) to come and sign (the hand card) for them. They are not paying us…it is a sacrifice. CHEW1.

### Appropriateness

The CHEWs and VHWs perceive that the innovation is increasing pregnant women’s contact with quality obstetric care and knowledge of danger signs in the obstetric period and addressing poor uptake of health facility-based obstetric services in the communities. It also increased the number of women accessing health facilities for ANC.

I went to a (pregnant) woman’s house… who didn’t bother about going to the hospital for antenatal care. I talked to her about going to the hospital… and I measured her blood pressure. She was excited. She said in her previous pregnancy, she went for antenatal care (in the hospital) and throughout her visits, her blood pressure wasn’t checked, VHW4

Everything (about implementing the innovation) works well. From the trainings to distributing the BP device, there is nothing else to add to the way the work is going. In fact, we have more (pregnant) women coming for ANC… which has increased our patient patronage, CHEW2.

The BP device (innovation) has helped because now (pregnant) women understand the danger of high blood pressure and its negative effects… now even if a woman is one month pregnant she will invite us to check her. This did not happen before introducing the BP device, VHW5

The CHEWs also perceive that the innovation has led to early identification and treatment of hypertensive disorders in pregnancy.

The pregnant woman (is able to) gets medication immediately. Once the VHW diagnosis her as having high BP, she comes to the health facility… immediately she gets her medication… not until a later stage of her sickness, CHEW3.

Most VHWs associate the appropriateness with the immediate value the BP device adds to their work, and the social acceptance it facilitates for them in the community.

When I did not have the CRADLE device, the (pregnant) did not value my worth. Now there is a CRADLE device added to the work I do, they respect me as a VHW. Pregnant women even refer their pregnant friends to me. They’d say, please help her doctor… they call me a doctor. I am happy. When I didn’t have the device, the clients barely gave me attention during home visits. They used to say I am disturbing them with the same health education message. Now they even offer me refreshments when I go to their homes, VHW6

### VHW’s competence with the BP device

The findings from the paired observation of VHW-client interaction shows that at the end line, 95% of the VHWs properly positioned the arm of the pregnant woman (baseline = 9%, *p* < 0.001), 95% properly applied cuff to the pregnant woman’s arm (baseline = 38%, *p* < 0.001), 99% waited to hear the beep of the device before pumping its bulb (baseline = 76%, *p* < 0.001), 99% stopped pumping the bulb after hearing the second beep from the device (baseline 75%, *p* < 0.001), 99% rightly identified the systolic and diastolic blood pressure readings (baseline = 94%, *p* = 0.04), 93% knew the three colors indicated by the device and the action to take (baseline = 84%, *p* = 0.04), and 82% could define the error messages of the device (baseline = 41%, *p* < 0.001) (Table 5).

**Table 5:** Result of paired observation of VHW-client interaction

Variable	Period	VHWs competent with using the device		
		*%, n = 118*	*95% CI*	*McNemar’s p value*
**Properly positons the arm of the client before applying the device**	Baseline	9	5–13	< 0.001
	End line	95	90–99	
**Properly applies the device’s cuff to the client’s arm**	Baseline	38	30–46	< 0.001
	End line	95	90–98	
**Hears the beep sound of the device before pumping the inflation bulb**	Baseline	76	67–84	< 0.001
	End line	99	98–100	
**Stops inflating the bulb after hearing the second beep sound**	Baseline	75	67–83	< 0.001
	End line	99	98–100	
**Can identify the BP readings on the device**	Baseline	94	90–98	0.04
	End line	99	98–100	
**Understands traffic color on the device and action to take**	Baseline	84	78–90	0.04
	End line	93	89–97	
**Can define error messages on the device**	Baseline	41	33–49	< 0.001
	End line	82	75–88	

The findings from the qualitative arm associate the improvement in VHWs’ competence with the refresher training and supportive supervision.

In the beginning we were trained, giving the device, and followed up to monitor how we were using the device and trained again…and our mistakes were corrected. After that, they (CHEWs) also continued to monitor our records and work… and correct our mistakes. I believe this made us do our work very well (properly). VHW3

### Prevalence of hypertensive disorder in pregnancy

The findings from the quantitative arm show a significant reduction in the prevalence of hypertension in pregnancy between the 1st and 2nd visits. The prevalence of hypertension in pregnancy dropped from 1.5 to 0.8% (*Z* = 4.04; *p* < 0.00001) between the two visits. We observed that the prevalence of isolated systolic hypertension in pregnancy (*Z* = 1.34, *p* = 0.18) and isolated diastolic hypertension in pregnancy (*Z* = 0.91, *p* = 0.36) was not affected between the two time points. The prevalence of isolated diastolic hypertension was highest during the first visit (8.5%; 95% CI 7.9–9) and second visit (8.1%; 95% CI 7.5–8.7). The prevalence of hypertensive disorder in the first visit was 13.8%. Of the 134 (100%) pregnant women with hypertension in pregnancy during the first visit, only 17 (13%) were yet to be normotensive by the second visit (Table 6).

**Table 6:** Prevalence of blood pressure disorder in pregnancy and impact of intervention

	First visit %, ***n*** = 9000 (95% CI)	Second visit %, ***N*** = 6992 (95%CI)	Test (***p*** value)
***Intervention’s effect on beneficiaries***			
**Hypertension in pregnancy**	1.5 (1.3–1.7)	0.8 (0.6–1)	*Z* = 4.04 (< 0.000001)
**Systolic hypertension only**	3.8 (3.4–4.2)	3.4 (3.0–3.8)	*Z* = 1.34 (0.18)
**Diastolic hypertension only**	8.5 (7.9–9.0)	8.1 (7.5–8.7)	*Z* = 0.91 (0.36)

The findings from the qualitative arm associate the drop in the prevalence of hypertension in pregnancy between the first and second VHW visits with an active referral of pregnant women with abnormal blood pressure to the local health facilities. Most VHWs expressed that sometimes, they accompany these cases to the hospital themselves.

There was an eight-month pregnant woman I visited. I measured her blood pressure (and) the indicator light showed a red… I told her to go to the hospital immediately. She was reluctant because she was not sure if the husband would give her permission. I insisted… and told her I’ll accompany her to the hospital. She called the husband on the phone… He then asked to speak with me. We spoke… and I explained everything to him. He immediately gave his wife the permission to go to the hospital. I’m glad I insisted. VHW7.

### Perceived barriers to sustainability of the innovation

The CHEWs believe that regular payment of the VHWs’ stipend is key to sustaining the innovation. They perceive that delayed or missed payment of VHWs’ stipend will affect the innovation.

… there will be a challenge with continuing the program if they stop the allowance. It is the reason (the stipend) why some of them (VHWs) are doing the work. CHEW SG

Even though they (VHWs) said they are volunteers working to improve their community and not because of money, but there is a need to continue to give them their incentive on time to improve their performance. CHEW3

Also, most CHEWs shared that the ongoing poor cooperation and participation of their colleagues in the local health facilities in implementing the intervention will hinder sustaining it. They expressed that the pushbacks from their colleagues could erode pregnant women’s confidence in the intervention.

When VHWs refer a client (pregnant woman) to the health facility… If the client takes the home visit card given to her by the VHW to the health facility and the person providing the healthcare refuses to fill it, she will perceive a negative feeling towards the program. She (the pregnant woman) will go tell other people that “I went to the facility and my card did not get signed or filled”. Such can pose a problem to the program. CHEW4.

Last, most CHEWs and VHWs express that drug stock out is an ongoing problem and that it will hinder scaling up the intervention. They indicate that the health facilities must be ready to cope with the increase in demand to treat hypertensive disorders in pregnancy.

There should be a constant supply of the lifesaving drugs, ANC drugs so that when we have high BP cases we will try our best… not that when they (cases with high BP) come, we keep telling them we don’t have the right drugs… they should go somewhere else. it will stop them from coming. CHEW5

## Discussion

In our study, we found that trained VHWs, i.e., level 1 CHWs can assess pregnant women’s blood pressure if equipped with proper technologies and increase early identification, diagnosis, and treatment of women with hypertension in pregnancy in resource-poor settings. We also found that the intervention and implementation approaches are appropriate and acceptable to stakeholders at the local levels. In Asia, level 1 CHWs already take part in assessing BP in non-pregnant populations [59, 60]. Our study provides evidence that these cadres of health workers can also assess the pregnant population’s BP with positive outcomes for the health system.

Our study also suggests that the intervention improved pregnant women’s health-seeking behavior and the health system’s continuum of care. Close to 14% of the pregnant women reached by the intervention had a hypertensive disorder during the VHWs’ first visit. By the VHWs’ second visit, 87% of those that had hypertension in pregnancy had become normotensive, suggesting that those referred by the VHWs sought care from the local health facilities. This corroborates the findings in other settings that show that CHW home visits during antenatal periods can improve health facility-based service use and outcomes of maternal and child health interventions [61, 62].

Also, our study suggests that level 1 CHWs are community resources for task-shifting strategies in resource-poor settings. The trained VHWs using the blood pressure-measuring device and house-to-house visits help to identify, refer, and follow up pregnant women at risk of pre-eclampsia and eclampsia. Overstretched health facility-based staff with inadequate access to accurate and reliable equipment to measure vital signs causes delayed treatment initiation for pre-eclampsia and eclampsia in resource-poor settings [20]. There are however policy issues to overcome in LMICs for level 1 CHWs to take part in managing hypertension in pregnancy [26, 28].

We found that the on-field refresher training and supportive supervisory role played by CHEWs are important for keeping the fidelity of the intervention. For instance, in our study, most VHWs could not use the Microlife blood pressure-monitoring device properly at baseline, despite training them in class. An on-field refresher training and follow-up supportive supervision by the CHEWs helped to improve the competence of the VHWs. The importance of the supervisory visits is corroborated by other studies that showed that supportive supervision and refresher training improve CHWs’ performance [22, 63].

We documented that isolated diastolic hypertension is the commonest hypertensive disorder in pregnancy among pregnant women in our study setting. This corroborates the findings from another multi-country study that showed unexpected high proportions of isolated diastolic hypertension among pregnant women using a similar BP-measuring device [64]. The diastolic blood pressure in early pregnancy is an important risk factor for pre-eclampsia and small for gestational age babies [65]. Between 9 and 30% of the isolated diastolic hypertension cases were later associated with systolic hypertension in the multi-country study [64].

Whether volunteering or salaried, sustaining financial incentives is key towards motivating CHWs to continue to deliver on their work [66]. We found in our study that delays in paying VHWs’ stipend can lead to demotivation and affect the intervention’s sustainability. Incentive-related “expectation gaps,” including lower than expected financial incentives and later than expected payments, influence VHWs’ motivation in many settings [22, 66].

Poor collaboration between CHEWs in a health facility has the potential to affect the scale-up of the intervention. Without effective collaboration between healthcare workers of the same cadre, providing effective care to patients in health facilities is difficult [67]. We put forward that it is important to de-emphasize individual roles in favor of team goals and collaboration when implementing the intervention in other settings [68].

A potential risk that our intervention poses is that it may reduce antenatal care services in health facilities if pregnant women believe they are already getting adequate antenatal care services at their “doorsteps.” The evidence however suggests that home visits by lay health workers who offer community-level antenatal care services to pregnant women do not reduce the use of obstetric services in health facilities in resource-poor settings [69]. This calls for the increased need to educate and encourage pregnant women to attend antenatal care despite the care they receive at home through the VHWs’ home visits.

### Strengths and limitation

A particular strength of our study lies in the theoretical underpinnings of our implementation approach. The REP framework provides an evidence-based structure for the implementation approach [51]. Also, the mixed method provided a broader understanding of the effect of the intervention and possibility of sustainability. One limitation of our study is that it is not an experimental study design. The true effect of the intervention on the prevalence of hypertensive disorders in pregnancy is better examined with a randomized control trial which may change if we control extraneous variables across the study settings [29]. Our study however increased early diagnosis and referral of cases of hypertension in pregnancy. Also, because we used VHWs for data collection, we kept the data element collected to a minimum. Reporting of false negatives is a concern when CHWs screen and diagnose diseases [70]. We used refresher training and regular supportive supervision to improve VHW competence and data collection during this study. We also did not collect information on gestational age of the pregnancy and treatment received from health facilities. This means that some pregnant women might have gotten assessed by the VHWs before 20 weeks of gestation, thus reducing the chances of identifying gestational hypertension in our study. Despite this, we expect only a small proportion of pregnant women with a gestational age of less than 20 weeks old in our study. This is because the evidence suggests that pregnant women in rural Africa keep their pregnancy a secret until it is about 20 weeks old [71–73]. We also could not determine the reason for the dropout from the study by the second VHW visits. The dropouts between the first and second visits in our study might have been cases of pregnancy loss, childbirth, relocation, or lost to follow-up for other reasons. Last, we did not collect data around why only 85% of the VHWs invited showed up for the VHW-client interaction. It is possible the 15% who did not show up were engaged in domestic farming activities at the time scheduled for the observation. Future studies should look into the limitations of our study to determine their potential for bias.

## Conclusion

Trained VHWs (level 1 CHWs) can assess pregnant women’s blood pressure if equipped with proper technologies. Also, they can increase early identification, diagnosis, and treatment of women with hypertension in pregnancy in resource-poor settings. The intervention and implementation approach based on the REP framework are appropriate and acceptable to stakeholders in resource-poor settings. The intervention will contribute to reducing maternal mortality and morbidity in low- and middle-income countries.

## Data Availability

Data for the study is in a secured database at the Society for Family Health. It can be assessed with a reasonable request to infor@sfh.org or janyanti@sfh.org.

## Abbreviations

VHW: Village health worker
CHW: Community health worker
CHEW: Community health extension worker
BP: Blood pressure
MNH: Maternal and newborn health
LGA: Local government area
REP: Replicating Effective Program
StaRI: Standards for Reporting Implementation Studies
COREQ: Consolidated Criteria for Reporting Qualitative Research
